# Knowledge, Attitude, and Practices Associated With COVID-19 Among Healthcare Workers in Hospitals: A Cross-Sectional Study in Saudi Arabia

**DOI:** 10.3389/fpubh.2021.643053

**Published:** 2021-07-23

**Authors:** Omar A. Almohammed, Leen A. Aldwihi, Adel M. Alragas, Ali I. Almoteer, Shivkumar Gopalakrishnan, Nasser M. Alqahtani

**Affiliations:** ^1^Department of Clinical Pharmacy, College of Pharmacy, King Saud University, Riyadh, Saudi Arabia; ^2^Pharmacy Department, King Saud University Medical City, Riyadh, Saudi Arabia; ^3^Department of Internal Medicine, Government Villupuram Medical College and Hospital, Mundiyampakkam, India; ^4^Riyadh First Health Cluster, Ministry of Health, Riyadh, Saudi Arabia

**Keywords:** COVID-19, health care workers, attitude, knowledge, practice, Saudi Arabia

## Abstract

Lack of knowledge among healthcare workers (HCWs) about infectious diseases leads to delayed diagnosis of new cases, spread of infection, and poor infection control practices. Therefore, HCWs based in hospitals must be equipped with good knowledge about the pathogen and disease to put up a robust fight against the virus. The aim of this study was to assess knowledge, attitude, and practices (KAP) of HCWs about coronavirus disease 2019 (COVID-19) at multiple public and private hospitals in Riyadh, Saudi Arabia. A cross-sectional, online questionnaire-based study was conducted between July and August of 2020. Logistic regression was used to investigate differences in the level of KAP among different participants. A total of 510 HCWs in hospitals completed the questionnaire. Only two-thirds of the participants (67.8%) had adequate knowledge about COVID-19, 72.2% of the participants had a positive attitude toward COVID-19, and 80.2% of the participants were practicing appropriately most of the time. Poor KAP was associated with a low education level. The females had better knowledge and attitude, whereas the males were more likely to practice appropriately most of the time. Notably, the participants from the nursing profession demonstrated a less favorable attitude compared with medical staff from other professions, but that did not prevent them from being the best when it comes to applying appropriate practices. The inadequate level of KAP among HCWs with the continuation of the pandemic and the possibility of a second wave demonstrates the need for continuous COVID-19-specific infection control training and emotional well-being supporting programs, especially for HCWs with a low education level.

## Introduction

Coronavirus disease 2019 (COVID-19) is the third and largest outbreak of coronavirus of this century (1). COVID-19 is caused by the novel coronavirus, namely, severe acute respiratory syndrome-coronavirus-2 (SARS-CoV-2). The first case in Saudi Arabia was reported on March 2, 2020 (2). There has been a relentless progression in numbers since day one after the first detection, despite the efforts being devoted to combating the pandemic situation. Till the day of finalizing this piece of research, there is no specific curative treatment, and few vaccines are available with limited supply; however, plenty of research and development activities are underway at national and international levels to explore a permanent curative treatment or vaccine to limit the impact of COVID-19 on the world.

In the absence of safe and effective treatment and a sufficient quantity of vaccines, enhanced awareness and infection control measures, along with other mitigation strategies, such as social distancing and face masks, are important tools in terms of COVID-19 pandemic management. Healthcare workers (HCWs) are at a higher risk than the general public in contracting the infection for multiple reasons, such as long working hours, suboptimal handwashing when meeting patients, and improper use of personnel protection equipment (PPE); in addition, this risk is not consistent among all HCWs as working in high-risk departments increases the risk of having the disease (3). The pivotal role of HCWs in pandemic control warrants good knowledge of infectious disease and hard work. Lack of knowledge and inadequate infection control training among HCWs delay diagnosis of new cases and are conducive to the spread of infection among HCWs (4). A retrospective national Saudi study revealed that 12.5% of all COVID-19 cases were among HCWs, and that 17.3% of these cases were asymptomatic (5). Additionally, HCW-related infections comprised 19.1% of Middle East respiratory syndrome (MERS) cases in Saudi Arabia (6). The reasons for infection transmission among HCWs include poor institutional infection control measures, lack of awareness or training, and poor compliance with PPE (7). Despite that COVID-19 has a lower fatality rate compared with MERS and SARS (8, 9), COVID-19 has resulted in more deaths than MERS and SARS combined.

The requirement of infection control training for all HCWs and the enforcement of strict infection control measures in hospitals can limit the spread of infection to HCWs and other hospitalized patients. After implementing strict infection control preparedness measures, a hospital in Hong Kong was able to have zero infection among HCWs, providing care for patients with COVID-19 over a 42-day observation period (10). Another large university hospital in Italy introduced infection prevention measures that were able to limit the spread of infection among HCWs to only 0.4% of employees over a 30-day observation period (11). In addition, the risk of being infected with SARS-CoV-2 was dependent on the ability of HCWs ability to follow implemented protective behavior measures, while contacts tracing among HCWs limited the spread of infection among HCWs (12). Furthermore, no nosocomial infection occurred in hospital wards characterized by higher biological risk and number of hospitalized patients with COVID-19. Instead, nosocomial infection occurred in areas with less hygiene practice (non-use of PPE and hand shaking), reflecting that low-risk perception results in higher rates of inappropriate practices among HCWs (12). In an English hospital, the incidence of COVID-19 among HCWs with direct patient contact was similar to those without, implying that community-acquired disease or transmission among HCWs was more likely than nosocomial transmission from patients (13). This implies that protective behavior measures were more likely to be followed in areas with high-risk perceptions. Furthermore, two studies examined the association between risk perception and adherence to protective behavior measures and found that the increase of risk perception to have the infection was an effective strategy to increase public adherence to protective measures (14, 15).

An acceptable level of knowledge about COVID-19 among HCWs was observed in multiple countries around the world (16–20). However, unreliable sources of information, such as social media, were used as the main source of information instead of more reliable sources (16, 17). Moreover, a study from China found an association between the level of knowledge and the level of attitude, whereas a Greece study concluded that the level of knowledge was associated with both the level of attitude and practice (16, 19). Therefore, the greater the knowledge of HCWs, the more confident they were in defeating the virus; and the low level of knowledge among HCWs was a major risk factor in failure in following infection control measures (16, 19). In addition, the lower level of education among HCWs was found to be associated with a lower level of knowledge about COVID-19 (20, 21).

As with any infectious disease, there is always a possibility of a second wave in which the number of cases increases again after transient decline (22, 23). A thorough review of published evidence suggests that pandemic situations throughout history, including the Spanish flu, came in waves, and higher rates of mortality were associated with subsequent waves (24–26). On the other hand, the second waves of more recent outbreaks, such as SARS and MERS, have been avoided due to the minimal severity of those outbreaks when compared with COVID-19 (27). However, a second wave of COVID-19 is highly expected and has already started in other regions of the world (23, 28). Individuals who do not fully understand the importance of preventive measures are always deemed a liability when it comes to the implementation of protective restrictions and protocols, social distancing, wearing of masks in public places, and practicing hand hygiene. This, in turn, puts more pressure on the medical staff.

Understanding the current level of knowledge and attitude about COVID-19 among HCWs based in hospitals and assessing their practices would help in the containment of the pandemic. Therefore, this study aimed to investigate the knowledge, attitude, and practices (KAP) of HCWs related to COVID-19 at work and in their personal life 4 months after the beginning of the pandemic in Saudi Arabia. The findings of this research will help us identify pitfalls and plan corrective measures to curb further outbreaks in healthcare institutions and the community.

## Materials and Methods

### Study Design and Subjects

A cross-sectional questionnaire-based study was conducted to assess KAP of HCWs regarding COVID-19. HCWs from multiple public and private hospitals providing care for hospitalized patients with COVID-19 in Riyadh, Saudi Arabia, were invited for participation. The KAP of the participants regarding COVID-19 was assessed, using an online questionnaire that was developed and scaled for this study. All workers in hospitals were eligible for participation in the study, including medical staff, such as physicians, nurses, pharmacists, laboratory technicians, and non-medical support staff in these hospitals, such as nonmedical administrators, security, maintenance, and housekeeping staff. All the participants gave their informed consent for inclusion in the study, and the study protocol was approved by the Institutional Review Board in the Riyadh First Health Cluster, Ministry of Health, Saudi Arabia (H1RI-29-Jul 17-01).

### Questionnaire

The questionnaire consisted of four parts (Appendix A). The first part included questions about the demographic characteristics of the participants (age, gender, marital status, highest educational level, being part of a medical or a nonmedical department, and the specific department for the medical staff), in addition to an item that inquired about the source of information of the participants about COVID-19. The age variable was assessed as a categorical variable where the participants were divided into five categories: 21–30 years, 31–40 years, 41–50 years, 51–60 years, and 61–70 years. Data on gender (female vs. male) and marital status (married vs. single) were captured and assessed as dichotomous variables. The highest educational level was assessed as a categorical level and broken down into four categories: high school or less, associate degree (diploma), bachelor or professional degree, postgraduate study or training. In addition, the participants were categorized based on their department or specialty as medical staff or non-medical supporting staff; and the medical staff were further broken down into medical or surgical, nursing, pharmacy, laboratory services, and other paraclinical services, such as physical therapy, respiratory therapy, radiology, etc.

The other three parts in the questionnaire included questions about the KAP of the participants KAP toward COVID-19. The knowledge section inquired about the etiology, epidemiology, pathogenesis, and management aspects of COVID-19. The attitude section gathered information about the fear of human-to-human transmission, endurance to emergency, personal commitment, responsibility, and optimism. The practice section included questions related to personal practices relating to appropriate usage of PPE at work, as well as the social and personal life of the participants.

Since no previous scales were available for this purpose, the questionnaire was constructed and approved by an international team from Saudi Arabia and India to ensure its face validity and to conduct the same study in both countries. The development of the questionnaire was mainly based on literature review and COVID-19 measures recommended by the World Health Organization and Ministry of Health. Six authors from the collaborative international team have individually revised the items for clarity and validity, and then it was revised by four researchers with experience in epidemiology, infectious diseases, and public health to ensure the appropriateness of the items and the validity of the scales. Overall, 10 researchers have individually revised the items in multiple rounds to ensure its internal validity. The questionnaire was then sent to a small group of respondents (*n* = 30) to pilot the questionnaire and take feedback on the items; minor changes were made at that time, and three items were removed from the initial questionnaire to improve the readability and clarity of the overall questionnaire. The final knowledge, attitude, and practice scales in the questionnaire had acceptable internal reliability with a Cronbach's α of 0.7, 0.6, and 0.8, respectively.

### Assessment of KAP of the Participants

For knowledge (nine items), the participants received 1 point for each correct answer and 0 point for incorrect answers. The participants with at least seven points were considered to have adequate knowledge about COVID-19. For attitude (eight items), the participants received two points for each positive response, one point for a neutral response, and zero point for a negative response. The participants with at least nine points were considered to have positive attitude. For practice (seven items), the participants received two points for “yes,” one point for “not sure,” and zero point for “no.” A minimum score of 11 points was considered to indicate safe or appropriate practice most of the time.

### Data Collection

Given the application of lockdown and social distancing measures during the time of the study, a convenience sample of HCWs from different departments was invited for participation through text messages or the chatting application “WhatsApp^®^.” At the end of the questionnaire, the participants were asked to share the link that was sent to them with their colleagues. Therefore, convenience and snowball sampling techniques were used in this study to avoid the disruption of the enforced social distancing measures.

The sent message had a link to the questionnaire, which could be completed on the cell phones of the participants. Data were collected over 5 weeks between July 1 and August 5, 2020, using an online survey tool for data collection (Microsoft Forms). The questionnaire was offered in Arabic and English for the convenience of the participants, and, when needed, the researchers were available for assistance in completing the questionnaire. The purpose of the questionnaire, voluntary participation and the anonymity of the respondents were clearly stated on the first page of the online questionnaire.

### Statistical Analysis

Descriptive statistics were used to present the demographics of the participants, frequencies of personnel with adequate or inadequate knowledge about COVID-19; positive, neutral, or negative attitude toward COVID-19; and application of safety practices for COVID-19. Since the accurateness of the scales for these concepts was not previously validated, the results from these scales should be considered as proxies for these concepts. Inferential statistics, the chi-Square test was used to investigate differences in the level of KAP among different participants, followed by Bonferroni *post hoc* tests, when necessary, to adjust for the effect of multiple groups comparison. Whereas multivariable logistic regression analysis was used to identify significant predictors for adequate knowledge, positive attitude, and appropriate practices while controlling for the effect of the other factors in the model. The Pearson correlation coefficient (r) was used to assess the association between the three scales in the study (knowledge, attitude, and practices toward COVID-19) in order to determine the relationship between these concepts under investigation. Assuming that 50% of the HCWs would have adequate knowledge about COVID-19, with a 95% confidence level and a 5% margin of error, the calculated sample size was estimated to be 385 subjects. An α level of <0.05 was used for statistical significance. The data were coded and analyzed, using SAS software (version 9.4; SAS Institute Inc., Cary, NC).

## Results

A total of 510 participants completed the questionnaire. Approximately half of the participants (52.3%) were between the ages of 21 and 30 years, and 27.3% were between the ages of 31 and 40 years. Women account for 53.7% of the participants, and 52.3% of all the participants were married. The majority of the participants (64.9%) had at least a bachelor or professional degree, and 17.4% had a high school diploma or less. In addition, the medical staff represented two-thirds of all HCWs participating in the study. A summary of participant characteristics is presented in Table 1. More than half of the study participants (52.7%) relied on multiple sources to obtain information about COVID-19, with the local ministry of health being the most common single source (23.3%). However, it is worth noting that only two-thirds of the study participants (66.5%) received at least one infection control training (Table 2).

**Table 1:** Characteristics of the participants.

**Characteristic**	**Number (%)**
**Age**	
21–30 years	267 (52.3)
31–40 years	139 (27.3)
41–50 years	69 (13.5)
≥ 51 years	35 (6.9)
**Gender**	
Male	236 (46.3)
Female	274 (53.7)
**Marital status**	
Single	243 (47.7)
Married	267 (52.3)
**Highest educational qualification**	
High school or less	89 (17.4)
Associate degree	90 (17.7)
Bachelor or professional degree	296 (58.0)
Postgraduate study or training (master, residency, or fellowship)	35 (6.9)
**Medical staff**	
No	137 (26.9)
Administrative staff	48 (9.4)
Supporting staff (security, maintenance, and housekeeping)	89 (17.5)
Yes	373 (73.1)
Medical or surgical	40 (10.7)
Pharmacy	59 (15.8)
Laboratory services	67 (18.0)
Nursing	89 (23.9)
Other para-clinical services	118 (31.6)

**Table 2:** Infection control training and main sources of information for COVID-19.

**Variable**	**Number (%)**
**Have you received any infection control training?**	
Yes	339 (66.5)
No	171 (33.5)
**The main sources of information for COVID-19**	
Relying mainly on a single source	241 (47.3)
Ministry of health	119 (23.3)
Social media	46 (9.0)
International health organization websites, like CDC and WHO	39 (7.7)
News media channels	37 (7.3)
Relying on multiple sources	269 (52.7)
All listed sources	91 (17.8)
International health organization websites and ministry of health	75 (14.7)
Ministry of health and social media	30 (5.9)
Less frequent combination of sources	73 (14.3)

### Knowledge

Overall, only two-thirds of the participants (67.8%) had adequate knowledge about COVID-19. Most were informed about the causative microbe for COVID-19 and the effectiveness of hand hygiene in eliminating it. In addition, 82.8% of the study participants knew that cough and fever were the most frequent symptoms experienced among infected individuals, and 85.7% of the participants recognized the actual method of transmission for the disease. Conversely, approximately half of the participants believed that there are multiple curative treatment options currently available for COVID-19. Moreover, 53.5% of the participants believed the pandemic would be over by summer, as the high temperature and humidity would destroy the virus. The knowledge scores were depicted in Table 3.

**Table 3:** Responses of the participants to the knowledge items on the questionnaire.

**Questionnaire items**	**Number (%)**
**1. COVID-19 is a contagious disease that is caused by?**	
a. Fungi	7 (1.3)
b. Bacteria	27 (5.2)
c. *Virus*	*459 (90.0)*
d. Parasite	2 (0.4)
e. I don't know	15 (2.9)
**2. The most common manifestation for the COVID-19 is?**	
a. *Cough and fever*	*422 (82.8)*
b. Stuffy and runny nose	23 (4.5)
c. Mild headache	25 (4.9)
d. Abdominal pain and diarrhea	22 (4.3)
e. I don't know	18 (3.5)
**3. The disease can easily spread through?**	
a. Food	28 (5.5)
b. Water	5 (1.0)
c. Close contact with animal	9 (1.7)
d. *Respiratory droplets and close contact*	*437 (85.7)*
e. I don't know	31 (6.1)
**4. What is the longest incubation period for COVID-19 before**	
**experiencing any symptoms?**	
a. 3 days	23 (4.5)
b. 5 days	31 (6.1)
c. 10 days	30 (5.9)
d. *14 days*	*368 (72.2)*
e. I don't know	58 (11.4)
**5. Severe cases and death are more common among?**	
a. Young children (<18 years)	14 (2.7)
b. Youth (19-30 years)	6 (1.2)
c. Older adults (31-60 years)	56 (11.0)
d. *Elderly (>60 years)*	*396 (77.7)*
e. I don't know	38 (7.4)
**6. Multiple proven curative treatment options are available now for COVID-19 all over the world?** ***(False)***	263 (51.6)
**7. Most COVID-19 cases are mild and can recover with no treatment?** ***(True)***	391 (76.7)
**8. We know that the pandemic will be over by summer, as the causative microbe is sensitive to high temperature and humidity?** ***(False)***	273 (53.5)
**9. Washing hands with soap and water is effective in eliminating the causative microbe**. ***(True)***	495 (97.1)

*Items 1–5 were multiple choice questions, whereas items 6–9 were true/false questions*.

*The choices in italic represent the correct answers for the item on the questionnaire*.

### Attitude

Overall, 72.2% of the participants had a positive attitude toward COVID-19 management. Most of the participants believed in the collaborative role of the community and healthcare system (88.6%) and the primary role of mass testing (83.9%) to successfully control the spread of the disease. Moreover, the majority of the participants (82%) believed that the country will contain the virus successfully. However, 78.6% expressed their fear or felt threatened when providing care for a patient with a suspected or confirmed case of COVID-19. Likewise, 56.5% of the responders believed the main reason for acquiring the infection by HCWs was negligence. Overall, the study participants showed favorable attitudes toward COVID-19 (Table 4).

**Table 4:** Responses of the participants to the attitude items on the questionnaire.

**Questionnaire items**	**Agree**	**Not Sure**	**Disagree**
1. In my opinion, all people in the healthcare system and the community are part of this battle against COVID-19, and should be responsible about their role.	452 (88.6)	49 (9.6)	9 (1.8)
2. I believe that early detection of COVID-19 cases through mass testing will facilitate or accelerate the control of the COVID-19 pandemic.	428 (83.9)	65 (12.8)	17 (3.3)
3. I think people who got infected with COVID-19, including health care personnel, were infected due to negligence.	184 (36.1)	104 (20.4)	222 (43.5)
4. I have a feel of threat or fear when I become close or provide care to a confirmed or suspected COVID-19 patient.	332 (65.1)	69 (13.5)	109 (21.4)
5. I think COVID-19 is just a communicable disease which is being given undue importance.	158 (31.0)	100 (19.6)	252 (49.4)
6. I think restricting travels, locking cities, and quarantining all suspected cases are an exaggeration for the current situation.	169 (33.1)	78 (15.3)	263 (51.6)
7. The country's efforts will succeed in the battle against COVID-19 pandemic.	418 (82.0)	78 (15.3)	14 (2.7)
8. I think when COVID-19 pandemic is over many benefits and good things will be seen.	403 (79.0)	68 (13.3)	39 (7.7)

*The responses in the table are represented as numbers (%)*.

*The underlined responses represent the positive attitude response for each item, and the not-sure responses represent the neutral attitude response*.

### Practices

Among all the participants, 80.2% reported complying with appropriate infection prevention and control practices most of the time. Approximately, 75% of HCWs donned PPE in the correct order, and 83.9% disposed of it appropriately most of the time. In addition, 80% of HCWs were avoided touching personal things while working and restricted their contact with people from external environments to limit the spread of the disease. Moreover, 89% of the study participants were willing to seek medical attention or advise others to do so when symptomatic. The practice items questionnaire and responses were summarized in Table 5.

**Table 5:** Responses of the participants to the practice items on the questionnaire.

**Which of the following practices you can say that you are practicing most or all of the time?**	**Yes**	**Not sure**	**No**
1. If I or anyone close to me develop any COVID-19 symptoms, I will seek or recommend to others to seek medical attention.	454 (89.0)	38 (7.5)	18 (3.5)
2. When I am putting on the personal protective equipment (PPE), I follow the following order: Suit—Mask—Goggles—Gloves	384 (75.3)	48 (9.4)	78 (15.3)
3. I have been careful not to carry my mobile phone/pen, etc.… inside the COVID-19 ward.	411 (80.6)	41 (8.0)	58 (11.4)
4. I don't go out unless it is necessary.	404 (79.2)	37 (7.3)	69 (13.5)
5. When I finish my shift, I dispose the PPE and scrub thoroughly before entering home/quarters.	453 (88.8)	38 (7.5)	19 (3.7)
6. I sanitize my hands with alcohol-based solution before attending to each patient.	455 (89.2)	45 (8.8)	10 (2.0)
7. After using my PPE, I dispose them in the appropriate color-coded bins.	428 (83.9)	53 (10.4)	29 (5.7)

*The responses in the Table are represented as numbers (%)*.

### KAP Based on the Demographics of the Participants

The proportions of HCWs with adequate knowledge differed by age (p <.001), and the participants younger than 30 years were more likely to have adequate knowledge than the participants from the age categories of 41–50 or older than 50 years (*p* < 0.0001), after adjusting for the effect of multiple groups comparison in the Bonferroni *post hoc* analysis. In addition, when compared with HCWs older than 40 years, the younger HCWs were more likely to follow appropriate practices most of the time. Besides that, the differences in the proportions of the participants with positive attitude from different age categories were not significant, after adjusting for the effect of multiple groups comparison. Moreover, the participants with higher levels of education documented adequate knowledge and positive attitude compared with the participants with lower levels of education (*p* < 0.001). In the *post hoc* analysis, this was significant when HCWs with an associate degree or less were compared with the participants with higher degrees and when the participants with high school or less were compared with HCWs with an associate degree. Besides that, only the participants with high school or less were less likely to follow appropriate practices most of the time. The results from the main analysis were presented in Table 6, whereas the results from the *post hoc* analysis were presented in Supplementary Tables 1A–6A.

**Table 6:** Distribution of adequate knowledge, positive attitude, and appropriate practices based on the demographics and characteristics of the participants.

**Variable**	**Adequate knowledge**		**Positive Attitude**		**Appropriate practices**	
	***N* (%)**	P^*^	**N (%)**	P^*^	***N* (%)**	P^*^
Overall (*n* = 510)	346 (67.8)	-	368 (72.2)	-	409 (80.2)	-
**Age**						
21–30 years	203 (76.0)	** <0.001**	207 (77.5)	**0.032**	229 (85.8)	** <0.001**
31–40 years	93 (66.9)		94 (67.6)		118 (84.9)	
41–50 years	35 (50.7)		43 (62.6)		44 (63.8)	
≥51 years	15 (42.9)		24 (68.6)		18 (51.4)	
**Gender**						
Male	151 (64.0)	0.087	162 (68.6)	0.100	201 (85.2)	**0.009**
Female	195 (71.2)		206 (75.2)		208 (75.9)	
**Marital status**						
Single	183 (75.3)	** <0.001**	187 (77.0)	**0.021**	205 (84.4)	**0.024**
Married	163 (61.1)		181 (67.8)		204 (76.4)	
**Educational achievement**						
High school or less	14 (15.7)	** <0.001**	31 (34.8)	** <0.001**	35 (39.3)	** <0.001**
Associate degree	52 (57.8)		59 (65.6)		80 (88.9)	
Bachelor or professional degree	249 (84.1)		245 (82.8)		262 (88.5)	
Postgraduate study or training	31 (88.6)		33 (94.3)		32 (91.4)	
**Received infection control training**						
No	115 (67.3)	0.839	132 (77.2)	0.072	115 (67.3)	** <0.001**
Yes	231 (68.1)		236 (69.6)		294 (86.7)	
**Medical staff**						
No	46 (33.6)	** <0.001**	70 (51.1)	** <0.001**	73 (53.3)	** <0.001**
Yes	300 (80.4)		298 (79.9)		336 (90.1)	
**Department for medical staff (*****n*** **=** **373)**						
Medical or surgical	34 (85.0)	** <0.001**	36 (90.0)	** <0.001**	33 (82.5)	**0.047**
Pharmacy	52 (88.1)		50 (84.8)		51 (86.4)	
Laboratory services	56 (83.6)		60 (89.6)		59 (88.1)	
Nursing	57 (64.0)		52 (58.4)		87 (97.8)	
Other para-clinical services	101 (85.6)		100 (84.8)		106 (88.1)	

*Values in bold represent significant results*.

* *P-values were from Chi-square test*.

Gender did not affect knowledge or attitude, but the males were more likely to adhere to appropriate practices compared with the females (*p* = 0.009). Moreover, single HCWs were more likely than married to have adequate knowledge, positive attitude, and follow appropriate practices most of the time. Interestingly, the attendance of infection control training did not affect knowledge or attitude but had a positive impact on practices among HCWs (86.7 vs. 67.3%; *p* < 0.001).

In addition, medical staff were more likely to exhibit adequate knowledge (80.4 vs. 33.6%; *p* < 0.001), positive attitude (79.9 vs. 51.1%; *p* < 0.001) and comply with appropriate infection prevention and control practices most of the time compared with non-medical support staff in the study (90.1 vs. 53.3%; *p* < 0.001). Among medical staff, nurses were less likely to have adequate knowledge compared with workers from pharmacy or other para-clinical services departments, and favorable attitude compared with workers from all other departments. However, that did not hinder nurses from following appropriate practices most of the time, especially when compared with HCWs from medical or surgical departments (*p* = 0.0017). The results from the main analysis were presented in Table 6, whereas the results from the *post hoc* analysis were presented in Supplementary Tables 7A–9A.

In the multivariable logistic regression, after adjusting for the interrelated effect of all variables on the KAP, some of the previous correlations have changed. Females were more likely to have adequate knowledge (AOR 1.86; 95% CI 1.20–3.00) and positive attitude (AOR 1.58; 95% CI 1.00–2.48) than males, but, when it comes to practice, they were less likely to follow appropriate practices (AOR 0.50; 95% CI 0.29–0.87). The level of educational achievement was a clear predictor for adequate knowledge, positive attitude, and appropriate practices; HCWs with higher educational achievements were more likely to have adequate knowledge, positive attitude, and practice appropriately most of the time compared with HCWs with lower educational achievement. As for the infection control training, HCWs who have attended these training did not have better knowledge (AOR 0.48; 95% CI 0.28–0.84), but they were more likely to have positive attitude (AOR 2.6; 95% CI 1.53–4.45) and practice appropriately most of the time (AOR 2.15; 95% CI 1.26–3.66) compared with HCWs who did not attend this training. The medical staff were more likely to have adequate knowledge (AOR 2.67; 95% CI 1.45–4.90) and comply with appropriate infection prevention and control practices most of the time (AOR 2.66; 95% CI 1.34–5.31) compared with non-medical support staff in the study. When controlled for all other variables, nursing staff did not have a lower level of knowledge than other medical staff, but they were much less likely to have a positive attitude than the medical and surgical staff. However, this low level of positive attitude did not hinder them from practicing appropriately as they were more likely to comply with appropriate infection prevention and control practices most of the time compared with the staff from the medical or surgical department (Table 7).

**Table 7:** Multivariable logistic regression for the adequate knowledge, positive attitude, and appropriate practices based on the demographics and characteristics of the participants.

**Variable**	**Adequate knowledge**	**Positive attitude**	**Appropriate practices**
	AOR (95%CI)^*^	**AOR (95%CI)**	**AOR (95%CI)**
**Age**			
21–30 years	Reference	Reference	Reference
31–40 years	0.88 (0.46–1.67)	0.78 (0.42–1.44)	0.86 (0.40–1.84)
41–50 years	0.65 (0.30–1.43)	0.90 (0.42–1.95)	0.51 (0.21–1.22)
≥51 years	0.69 (0.24–2.04)	1.95 (0.70–5.46)	0.35 (0.12–1.06)
**Gender**			
Male	Reference	Reference	Reference
Female	**1.86 (1.20–3.00)**	**1.58 (1.00–2.48)**	**0.50 (0.29–0.87)**
**Marital status**			
Single	Reference	Reference	Reference
Married	0.80 (0.44–1.46)	0.72 (0.40–2.48)	1.29 (0.64–2.59)
**Educational achievement**			
High school or less	Reference	Reference	Reference
Associate degree	**4.43 (1.85**–**10.61)**	**4.76 (2.06**–**11.01)**	**5.69 (2.20**–**14.75)**
Bachelor or professional degree	**16.79 (7.47**–**37.74)**	**11.10 (5.10**–**24.18)**	**4.60 (2.17**–**9.75)**
Postgraduate study or training	**36.09 (9.46**–**137.7)**	**52.6 (10.31**–**268.08)**	**6.02 (1.47**–**24.59)**
**Received infection control training**			
No	Reference	Reference	Reference
Yes	**0.48 (0.28**–**0.84)**	**2.6 (1.53**–**4.45)**	**2.15 (1.26**–**3.66)**
**Medical staff**			
No	Reference	Reference	Reference
Yes	**2.67 (1.45**–**4.90)**	1.30 (0.69–2.48)	**2.66 (1.34**–**5.31)**
**Department for medical staff (*****n*** **=** **373)^†^**			
Medical or surgical	Reference	Reference	Reference
Pharmacy	2.06 (0.56–7.63)	0.97 (0.25–3.67)	1.49 (0.43–5.24)
Laboratory services	0.83 (0.25–2.76)	1.21 (0.31–4.77)	2.08 (0.63–6.88)
Nursing	0.55 (0.18–1.67)	**0.27 (0.08**–**0.90)**	**12.52 (2.56**–**69.55)**
Other para-clinical services	1.19 (0.38–3.70)	0.78 (0.23–2.67)	2.77 (0.92–8.39)

*AOR, adjusted odds ratio, 95% CI, 95% confidence interval. Values in bold represent significant results*.

* *The multivariable logistic is adjusted for age, gender, marital status, educational achievement, being part of the medical staff and receiving infection control training*.

† *The comparison is adjusted for age, gender, marital status, educational achievement, and receiving infection control training*.

The results of the correlation analysis revealed a positive and moderate correlation between the knowledge and attitude scales (*r* =.53; *p* <.001) and between the knowledge and practice scales (*r* = 0.51; *p* < 0.001). Whereas the relationship between the attitude and practice scales was positive but weak (*r* = 0.34; *p* < 0.001). The results from the correlation analysis are presented in Table 8.

**Table 8:** The correlation between the knowledge, attitude, and practice scales.

**Scales**	**Knowledge**	**Attitude**	**Practice**
**Knowledge**		*r* = 0.53	*r* = 0.51
		***P*** **<** **0.001**	***P*** **<** **0.001**
**Attitude**			*r* = 0.34
			***P*** **<** **0.001**
**Practice**			

*Values in bold represent significant results*.

## Discussion

The COVID-19 pandemic has caused the loss of innumerable lives and paralyzed the economy of many countries globally (29). According to the Ministry of Health update from October 19, 2020, the number of COVID-19 cases was ~342,000 in Saudi Arabia (30). For a disease without curative treatment, prevention is the only solution. Until the majority of the population becomes vaccinated and the country reaches the needed level of herd immunity, preventive measures rely heavily upon the KAP of the population affected. In nations affected by the pandemic, health systems should house a robust workforce and not only medical equipment. The knowledge and behavior of HCWs are of crucial importance in preventing transmission. If neglected, the HCWs could endanger their own life, in addition to making themselves potential vectors (3). We timed this study in the middle stages of the COVID-19 outbreak in Saudi Arabia with the purpose of investigating the KAP of HCWs as it relates to the management of COVID-19 at work in hospitals and in their personal life. Approximately two-thirds of HCWs (67.8%) had adequate knowledge, 72.2% had a positive attitude, and 80.2% were practicing precautionary behaviors. Only 66.5% of the participants received infection control training. After controlling for the effect of other variables, non-medical staff had a lower level of knowledge compared with medical staff, and the KAP were highly dependent on the levels of education of HCWs. Moreover, the nursing staff had comparatively less favorable attitudes toward COVID-19 but were more likely to comply with appropriate practices compared with medical staff from other professions.

The current study revealed that most participants had adequate knowledge about the nature of the illness (90%), common manifestations (82.8%), the mode of spread (85.7%), and preventive measures (97.1%). However, only 72.3% were aware of the incubation period of COVID-19. This leaves an important gap in the knowledge, which might result in disregarding the isolation rules by HCWs. Although strong evidence was lacking regarding the possibility of COVID-19 pandemic suppression by high temperatures and humidity and confirmed COVID-19 cases have been reported in regions with hot or humid climates, such as the African equatorial region, the Amazon rainforest regions, and the Middle East (31), myths still flourish among HCWs as sizeable 53.5% of the participants believed that the pandemic would be over by summer. These important deficiencies in knowledge are probably owing to a lack of scientific updating at both individual and community levels, as well as the lack of training programs. HCWs are burdened by their pandemic work schedule, which, therefore, mandates the intervention of administrative authorities to ensure periodic training for them.

Multiple studies conducted around the world have documented the average level of knowledge among HCWs to be >80% [China (16), Uganda (17), and Pakistan (18)]. The knowledge level in our study participants was strikingly less than that of the world average. A probable explanation for this observed phenomenon could be the mixed study population comprised of both medical and non-medical HCWs based in hospitals. Moreover, the findings of this study are consistent with those of Zhang et al., who reported that medical staff in China had sufficient knowledge about COVID-19 and indicated that physicians showed higher knowledge compared with nurses or paramedics (16). In addition, regional studies conducted in Greece found that the vast majority of medical staff were informed about COVID-19, but there were inconsistencies in responses related to the transmission of the disease (19). Interestingly, the current study results parallel to those of Nemati et al., who reported that only 56.5% of Iranian nurses had sufficient knowledge about transmission, symptoms, and treatment of COVID-19 (32).

In terms of attitude, the majority of the study participants showed a favorable profile. Most of the participants have imposed faith on the ability of the government to control the disease (82%). This is in line with a previous study from Pakistan that highlighted similar results (18). Previously, Olum et al. suggested that medical staff exhibited poor attitudes concerning the SARS-CoV-2 pandemic (17). Our study revealed a positive attitude prevalent among HCWs, which is probably attributable to high motivation levels and proper administrative support from policymakers. Our study also highlighted that adequate knowledge correlated well with a positive attitude among HCWs. These findings echoed those of previous studies, in which good levels of knowledge among HCWs resulted in a positive attitude regarding COVID-19 (16, 18, 20).

In the practice domain, HCWs based in hospitals demonstrated good adherence to safety practices most of the time (80.2%). This was reflected in the frequency of unnecessary leaves taken, proper usage of PPE and frequency of handwashing. It is encouraging to compare this figure with that reported by Saqlain et al., who found that 88.7% of medical staff followed a good practice to control the disease (18). Our figure is marginally lower than the estimate of Saqlain et al. but was much higher than that of Olum et al., thereby proving wide regional variation in practices. Likewise, our study participants did not share congruence in practice with the results reported by Papagiannis et al., whose respondents reported that they washed hands often or very often, and that 24.9% washed hands before and after contact with the patient or environment of the patient (19). This aspect of practice showed wide variations internationally because it depended not only on knowledge but also on adequate water supply and infrastructure.

Although the difference in age and marital status was initially a significant predictor for the KAP, when we adjusted for the effect of other variables in the study, this became an insignificant predictor. Females were more likely to have adequate knowledge and a positive attitude toward COVID-19, but males were more likely to comply with appropriate practices about COVID-19. Moreover, HCWs with a higher level of education and from the medical staff were more likely to have adequate knowledge and practice appropriately in regard to COVID-19 than HCWs with a lower level of education and non-medical staff in hospitals. These findings were similar to that from Hubei, China where they found male residents to have a lower level of knowledge than females and a lower level of education to be associated with a lower level of knowledge about COVID-19 (21). Typically, infection control training is recommended or required on medical staff in hospitals, who are usually highly educated compared with other non-medical HCWs in hospitals. This is very important in the case of COVID-19, as our findings indicate that these HCWs with a lower level of education and training might be at higher risk of contracting the disease or spreading it to others in their institutions or residence. As the finding from this study and previous studies indicated (20, 21), HCWs with higher educational achievement were more likely to have adequate knowledge about COVID-19, probably owing to these capabilities of HCWs to find the right source of information, such as published research and good training programs. Therefore, providing HCWs with lower educational qualification, especially non-medical staff, with specially designed COVID-19 training sessions, is highly encouraged to fill the gap in knowledge and practice between them and the rest of the HCWs.

After controlling for the effect of other variables in the study, no significant difference was seen among HCWs from the medical staff working in different departments, except for the nursing staff who were less likely to have a positive attitude toward COVID-19, but that did not prevent them from being the best department when it comes to applying appropriate practices most of the time. Since only two thirds of the participants in the study have received any infection control training, this indicates that these training programs were not required for all HCWs in hospitals. Although HCWs who previously participated in infection control training did not have better knowledge about COVID-19 than the one who did not, they were more likely to have a positive attitude toward COVID-19 and follow appropriate infection control practices most of the time. The study did not specify the type of infection control training the HCWs received or when they received it. Thus, this infection control training could have been from the general training sessions that did not have any aspect about COVID-19. Usually, infection control training would improve the KAP of the participants about infections and infection control practices (33–35), but, in our case, the knowledge items on the questionnaire were specific about COVID-19, which might explain why participation in such training did not impact the knowledge of the participants about COVID-19. Therefore, HCWs may need to receive distinct infection control training programs about COVID-19, and these sessions could be provided once with follow-up materials on the updates.

The major limitation of this study is that the design is descriptive in nature, which fails to establish the causal relationship. No specific theory was used in the study; thus, the findings can only be explained by speculation. No previously validated tools assessing KAP about COVID-19 among HCWs in hospitals have determined a cutoff point for adequate knowledge, positive attitude, and an appropriate level of practice at the time of the study; therefore, the authors have predefined a cutoff point for the study purposes. With that in mind, the findings from the scales in this study should be interpreted as proxies for these concepts, and the need for validated scales to measure KAP associated with COVID-19 still existing. Although the use of a three-level scale to capture variation in any concept is the least reliable, it was deemed appropriate by the researchers in this case as it minimizes the burden on HCWs when completing the questionnaire and the risk of discontinuing response to items on the questionnaire for tiredness or busyness. Moreover, relative lack of nationwide research studies investigating this field hampers comparison and makes our discussion limited. One more methodological limitation is that the study used convenience and snowball sampling techniques, which limits our ability to calculate the response rate in the study. Therefore, the study external validity was negatively impacted, and the results from this study cannot be generalized to other HCWs. Although the questionnaire was offered in two languages and the researchers were available to offer help in explaining questions and options when needed, most of the participants completed the questionnaire without assistance. The offering of the questionnaire in two languages and availability of researchers for assistance were mainly to be inclusive for non-Arabic speakers in practice. In addition, <10% of the participants needed assistance in completing the questionnaire, which we do not believe have impacted our results. The use of an online questionnaire without asking for identifiers of the participants should have ensured the anonymity and confidentiality of the participants to minimize the risk of social desirability.

The study identified adequate knowledge among two-thirds of HCWs based in hospitals, which was less than international levels, but the attitude and practice aspects were parallel to most other countries. The knowledge deficit and poor training status warrant a revamp of training programs for HCWs in hospitals. In addition, the study identified a vulnerable target group of HCWS who have not received any infection control training and the ones with a low level of education. This group of HCWs needs special attention through mandatory enrollment in infection control training programs in order to protect them at work and to protect people working or living around them from having COVID-19 or any other infectious diseases that may spread in these hospitals. This comes in the light of the fact that improvement in knowledge positively influences attitudes and practice. The authors recommend continuous professional development programs for all HCWs in hospitals targeting heightened awareness and supporting programs to maintain the emotional well-being for HCWs with the continuation of the pandemic. Finally, the directors of hospitals should take responsibility for offering these training and supporting programs and keep track of employee enrollment. This might protect the employees, limit the number of sick leave days, and create a better work environment.

## Data Availability Statement

The raw data supporting the conclusions of this article will be made available by the authors, without undue reservation.

## Ethics Statement

The studies involving human participants were reviewed and approved by Institutional Review Board in the Riyadh First Health Cluster, Ministry of Health, Saudi Arabia (H1RI-29-Jul17-01). The participants provided their written informed consent to participate in this study.

## Author Contributions

OA and SG: conceptualization. OA: methodology, software, formal analysis, supervision, project administration, and funding acquisition. SG and LA: validation. AMA and AIA: investigation. LA: data curation. AMA and LA: writing—original draft preparation. NA and SG: writing—review and editing and visualization. All authors have read and agreed to the published version of the manuscript.

## Conflict of Interest

The authors declare that the research was conducted in the absence of any commercial or financial relationships that could be construed as a potential conflict of interest.

## References

[1] Peeri NC Shrestha N Rahman MS Zaki R Tan Z Bibi S. The SARS, MERS and novel coronavirus (COVID-19) epidemics, the newest and biggest global health threats: what lessons have we learned? Int J Epidemiol. (2020) 49:717–26. 10.1093/ije/dyaa033 32086938 PMC7197734

[2] Ministry of Health in Saudi Arabia. MOH reports first case of coronavirus infection. (2020) Available online at: https://www.moh.gov.sa/en/Ministry/MediaCenter/News/Pages/News-2020-03-02-002.aspx (accessed June 27, 2020).

[3] Chou R Dana T Buckley DI Selph S Fu R Totten AM. Epidemiology of and risk factors for coronavirus infection in health care workers: a living rapid review. Ann Intern Med. (2020) 173:120–36. 10.7326/M20-1632 32369541 PMC7240841

[4] Suwantarat N Apisarnthanarak A. Risks to healthcare workers with emerging diseases: lessons from MERS-CoV, Ebola, SARS, and avian flu. Curr Opin Infect Dis. (2015) 28:349–61. 10.1097/QCO.0000000000000183 26098498

[5] Alsofayan YM Althunayyan SM Khan AA Hakawi A Assiri AM. Clinical characteristics of COVID-19 in Saudi Arabia: a national retrospective study. J Infect Public Health. (2020) 13:920–25. 10.1016/j.jiph.2020.05.026 32534945 PMC7833063

[6] Xiao J Fang M Chen Q He B. SARS, MERS and COVID-19 among healthcare workers: a narrative review. J Infect Public Health. (2020) 13:843–48. 10.1016/j.jiph.2020.05.019 32493671 PMC7250777

[7] Al-Tawfiq JA Auwaerter PG. Healthcare-associated infections: the hallmark of Middle East respiratory syndrome coronavirus with review of the literature. J Hosp Infect. (2019) 101:20–9. 10.1016/j.jhin.2018.05.021 29864486 PMC7114594

[8] Mahase E. Coronavirus: covid-19 has killed more people than SARS and MERS combined, despite lower case fatality rate. BMJ. (2020) 368:m641. 10.1136/bmj.m641 32071063

[9] Petrosillo N Viceconte G Ergonul O Ippolito G Petersen E. COVID-19, SARS and MERS: are they closely related? Clin Microbiol Infect. (2020) 26:729–34. 10.1016/j.cmi.2020.03.026 32234451 PMC7176926

[10] Cheng VCC Wong S-C Chen JHK Yip CCY Chuang VWM Tsang OTY. Escalating infection control response to the rapidly evolving epidemiology of the coronavirus disease 2019 (COVID-19) due to SARS-CoV-2 in Hong Kong. Infect Control Hosp Epidemiol. (2020) 41:493–98. 10.1017/ice.2020.58 32131908 PMC7137535

[11] Vimercati L Dell'Erba A Migliore G De Maria L Caputi A Quarato M. Prevention and protection measures of healthcare workers exposed to SARS-CoV-2 in a university hospital in Bari, Apulia, Southern Italy. J Hosp Infect. (2020) 105:454–58. 10.1016/j.jhin.2020.05.024 32445776 PMC7239006

[12] Vimercati L De Maria L Quarato M Caputi A Stefanizzi P Gesualdo L. COVID-19 hospital outbreaks: protecting healthcare workers to protect frail patients. An Italian observational cohort study. Int J Infect Dis. (2021) 102:532–37. 10.1016/j.ijid.2020.10.098 33157297 PMC7610093

[13] Hunter E Price DA Murphy E van der Loeff IS Baker KF Lendrem D . First experience of COVID-19 screening of health-care workers in England. Lancet. (2020) 395:e77–8. 10.1016/S0140-6736(20)30970-3 32333843 PMC7176380

[14] Duan T Jiang H Deng X Zhang Q Wang F. Government intervention, risk perception, and the adoption of protective action recommendations: evidence from the COVID-19 prevention and control experience of China. Int J Environ Res Public Health. (2020) 17:3387. 10.3390/ijerph17103387 32414013 PMC7277925

[15] Bruine de Bruin W Bennett D. Relationships between initial COVID-19 risk perceptions and protective health behaviors: a national survey. Am J Prev Med. (2020) 59:157–67. 10.1016/j.amepre.2020.05.001 32576418 PMC7242956

[16] Zhang M Zhou M Tang F Wang Y Nie H Zhang L. Knowledge, attitude, and practice regarding COVID-19 among healthcare workers in Henan, China. J Hosp Infect. (2020) 105:183–87. 10.1016/j.jhin.2020.04.012 32278701 PMC7194961

[17] Olum R Chekwech G Wekha G Nassozi DR Bongomin F. Coronavirus disease-2019: knowledge, attitude, and practices of health care workers at Makerere university teaching hospitals, Uganda. Front Public Health. (2020) 8:181. 10.3389/fpubh.2020.00181 32426320 PMC7204940

[18] Saqlain M Munir MM Rehman SU Gulzar A Naz S Ahmed Z. Knowledge, attitude, practice and perceived barriers among healthcare workers regarding COVID-19: a cross-sectional survey from Pakistan. J Hosp Infect. (2020) 105:419–23. 10.1016/j.jhin.2020.05.007 32437822 PMC7211584

[19] Papagiannis D Malli F Raptis DG Papathanasiou IV Fradelos EC Daniil Z. Assessment of knowledge, attitudes, and practices towards new coronavirus (SARS-CoV-2) of health care professionals in Greece before the outbreak period. Int J Environ Res Public Health. (2020) 17:4925. 10.3390/ijerph17144925 32650614 PMC7400230

[20] Kassie BA Adane A Tilahun YT Kassahun EA Ayele AS Belew AK. Knowledge and attitude towards COVID-19 and associated factors among health care providers in Northwest Ethiopia. PLoS ONE. (2020) 15:e0238415. 10.1371/journal.pone.0238415 32857811 PMC7454942

[21] Zhong B-L Luo W Li H-M Qian-Qian Zhang Q-Q Liu X-G Li W-T. Knowledge, attitudes, and practices towards COVID-19 among Chinese residents during the rapid rise period of the COVID-19 outbreak: a quick online cross-sectional survey. Int J Biol Sci. (2020) 16:1745–52. 10.7150/ijbs.45221 32226294 PMC7098034

[22] Cacciapaglia G Cot C Sannino F. Second wave COVID-19 pandemics in Europe: a temporal playbook. Sci Rep. (2020) 10:15514. 10.1038/s41598-020-72611-5 32968181 PMC7511360

[23] Mahase E. Covid-19: UK government must “get its act together” as modelling suggests 85 000 deaths in second wave, experts say. BMJ. (2020). 10.1136/bmj.m4242. [Epub ahead of print]. 33127605

[24] Smith D. Pandemic risk modelling. In: The Palgrave Handbook of Unconventional Risk Transfer. Cham: Palgrave Macmillan (2017). p. 463–495. 10.1007/978-3-319-59297-8_15

[25] Mummert A Weiss H Long L-P Amigó JM Wan X-F. A perspective on multiple waves of influenza pandemics. PLoS ONE. (2013) 8:e60343. 10.1371/journal.pone.0060343 23637746 PMC3634039

[26] Miller MA Viboud C Balinska M Simonsen L. The signature features of influenza pandemics—implications for policy. NEJM. (2009) 360:2595–98. 10.1056/NEJMp0903906 19423872

[27] Ghanbari B. On forecasting the spread of the COVID-19 in Iran: the second wave. Chaos Solitons Fractals. (2020) 140:110176. 10.1016/j.chaos.2020.110176 32834656 PMC7386426

[28] Wise J. Covid-19: risk of second wave is very real, say researchers. BMJ. (2020) 369:m2294. 10.1136/bmj.m2294 32518177

[29] Nicola M Alsafi Z Sohrabi C Kerwan A Al-Jabir A Iosifidis C. The socio-economic implications of the coronavirus pandemic (COVID-19): a review. Int J Surg. (2020) 78:185–93. 10.1016/j.ijsu.2020.04.018 32305533 PMC7162753

[30] Ministry of Health in Saudi Arabia. COVID 19 Dashboard: Saudi Arabia. Available online at: https://covid19.moh.gov.sa/ (accessed October 19, 2020).

[31] Wu Y Jing W Liu J Ma Q Yuan J Wang Y. Effects of temperature and humidity on the daily new cases and new deaths of COVID-19 in 166 countries. Sci Total Environ. (2020) 729:139051. 10.1016/j.scitotenv.2020.139051 32361460 PMC7187824

[32] Nemati M Ebrahimi B Nemati F. Assessment of Iranian nurses' knowledge and anxiety toward COVID-19 during the current outbreak in Iran. Arch Clin Infect Dis. (2020) 15:e102848. 10.5812/archcid.102848

[33] Yousefi H Nahidian M Sabouhi F. Reviewing the effects of an educational program about sepsis care on knowledge, attitude, and practice of nurses in intensive care units. Iran J Nurs Midwifery Res. (2012) 17:S91–5. https://www.ncbi.nlm.nih.gov/pmc/articles/PMC3696963/23833608 PMC3696963

[34] Galal YS Labib JR Abouelhamd WA. Impact of an infection-control program on nurses' knowledge and attitude in pediatric intensive care units at Cairo University hospitals. J Egypt Public Health Assoc. (2014) 89:22–8. 10.1097/01.EPX.0000444562.71691.06 24717397

[35] Attari A Aminoroaia M Maracy MR. Efficacy of purposeful educational workshop of medical and nonmedical interventions based on needs assessments in nurses. J Educ Health Promot. (2017) 6:18. 10.4103/2277-9531.204745 28546983 PMC5433647

