# Corrigendum: Knowledge, Attitude, and Practices Associated With COVID-19 Among Healthcare Workers in Hospitals: A Cross-Sectional Study in Saudi Arabia

**DOI:** 10.3389/fpubh.2022.863354

**Published:** 2022-03-07

**Authors:** Omar A. Almohammed, Leen A. Aldwihi, Adel M. Alragas, Ali I. Almoteer, Shivkumar Gopalakrishnan, Nasser M. Alqahtani

**Affiliations:** ^1^Department of Clinical Pharmacy, College of Pharmacy, King Saud University, Riyadh, Saudi Arabia; ^2^Pharmacy Department, King Saud University Medical City, Riyadh, Saudi Arabia; ^3^Department of Internal Medicine, Government Villupuram Medical College and Hospital, Mundiyampakkam, India; ^4^Riyadh First Health Cluster, Ministry of Health, Riyadh, Saudi Arabia

**Keywords:** COVID-19, health care workers, attitude, knowledge, practice, Saudi Arabia

There is an error in the **Funding** statement. The correct name for the funder is “Researcher Supporting Project (RSP-2020/77), King Saud University, Riyadh, Saudi Arabia.” The name was correct in the **Funding** section but incorrect in the **Acknowledgments** section as an additional statement was mistakenly added to the **Acknowledgments** section.

The authors apologize for this error and state that this does not change the scientific conclusions of the article in any way. The original article has been updated.

## Publisher's Note

All claims expressed in this article are solely those of the authors and do not necessarily represent those of their affiliated organizations, or those of the publisher, the editors and the reviewers. Any product that may be evaluated in this article, or claim that may be made by its manufacturer, is not guaranteed or endorsed by the publisher.

