# Supplementary material for: Knowledge, Attitude, and Practices Associated With COVID-19 Among Healthcare Workers in Hospitals: A Cross-Sectional Study in Saudi Arabia
Source: Front Public Health. 2021 Jul 23;9:643053. doi: 10.3389/fpubh.2021.643053 (PMC8342857; doi:10.3389/fpubh.2021.643053)
Supplement: Supplementary file 1 [file Table_1.DOCX]

| Table 1A. Bonferroni post hoc analysis for the adjusted comparison between participants in different age categories in terms of having adequate knowledge | | | | |
| --- | --- | --- | --- | --- |
| Age categories | 21 – 30 years | 31 – 40 years | 41 – 50 years | ≥ 51 years |
| 21 – 30 years |  | 76.0% vs. 66.9% | 76.0% vs. 50.7% | 76.0% vs. 42.9% |
| 31 – 40 years | 0.0497^*^ |  | 66.9% vs. 50.7% | 66.9% vs. 42.9% |
| 41 – 50 years | **<0.0001** | 0.0239 |  | 50.7% vs. 42.9% |
| ≥ 51 years | **<0.0001** | 0.0088 | 0.4480 |  |
| Data above the diagonal line represent the comparison in terms of proportions of patients with adequate knowledge in the two categories under investigation. Data beneath the diagonal line represent the *p*-values from the Bonferroni post hoc analysis for each two categories under investigation.  ^*^ *p*-values <0.0083 from Bonferroni post hoc analysis is considered statistically significant, and cells with numbers in bold represent comparisons with significant differences between the two categories under investigation. | | | | |

**Appendix B**

| Table 2A. Bonferroni post hoc analysis for the adjusted comparison between participants in different age categories in terms of having positive attitude | | | | |
| --- | --- | --- | --- | --- |
| Age categories | 21 – 30 years | 31 – 40 years | 41 – 50 years | ≥ 51 years |
| 21 – 30 years |  | 77.5% vs. 67.6% | 77.5% vs. 62.6% | 77.5% vs. 68.6% |
| 31 – 40 years | 0.0306^*^ |  | 67.6% vs. 62.6% | 67.6% vs. 68.6% |
| 41 – 50 years | 0.0099 | 0.4472 |  | 62.6% vs. 68.6% |
| ≥ 51 years | 0.2400 | 0.9148 | 0.5291 |  |
| Data above the diagonal line represent the comparison in terms of proportions of patients with adequate knowledge in the two categories under investigation. Data beneath the diagonal line represent the *p*-values from the Bonferroni post hoc analysis for each two categories under investigation.  ^*^ *p*-values <0.0083 from Bonferroni post hoc analysis is considered statistically significant, and cells with numbers in bold represent comparisons with significant differences between the two categories under investigation. | | | | |

| Table 3A. Bonferroni post hoc analysis for the adjusted comparison between participants in different age categories in terms of complying with appropriate practices most of the time | | | | |
| --- | --- | --- | --- | --- |
| Age categories | 21 – 30 years | 31 – 40 years | 41 – 50 years | ≥ 51 years |
| 21 – 30 years |  | 85.8% vs. 84.9% | 85.8% vs. 63.8% | 85.8% vs. 51.4% |
| 31 – 40 years | 0.8122^*^ |  | 84.9% vs. 63.8% | 84.9% vs. 51.4% |
| 41 – 50 years | **<0.0001** | **0.0005** |  | 63.8% vs. 51.4% |
| ≥ 51 years | **<0.0001** | **<0.0001** | 0.2256 |  |
| Data above the diagonal line represent the comparison in terms of proportions of patients with adequate knowledge in the two categories under investigation. Data beneath the diagonal line represent the *p*-values from the Bonferroni post hoc analysis for each two categories under investigation.  ^*^ *p*-values <0.0083 from Bonferroni post hoc analysis is considered statistically significant, and cells with numbers in bold represent comparisons with significant differences between the two categories under investigation. | | | | |

| Table 4A. Bonferroni post hoc analysis for the adjusted comparison between participants from different levels of education in terms of having adequate knowledge | | | | |
| --- | --- | --- | --- | --- |
| Level of education | High school or less | Associate degree | Bachelor or professional degree | Postgraduate study or training |
| High school or less |  | 15.7% vs. 57.8% | 15.7% vs. 84.1% | 15.7% vs. 88.6% |
| Associate degree | **<0.0001**^*^ |  | 57.8% vs. 84.1% | 57.8% vs. 88.6% |
| Bachelor or professional degree | **<0.0001** | **<0.0001** |  | 84.1% vs. 88.6% |
| Postgraduate study or training | **<0.0001** | **0.0011** | 0.4905 |  |
| Data above the diagonal line represent the comparison in terms of proportions of patients with adequate knowledge in the two categories under investigation. Data beneath the diagonal line represent the *p*-values from the Bonferroni post hoc analysis for each two categories under investigation.  ^*^ *p*-values <0.0083 from Bonferroni post hoc analysis is considered statistically significant, and cells with numbers in bold represent comparisons with significant differences between the two categories under investigation. | | | | |

| Table 5A. Bonferroni post hoc analysis for the adjusted comparison between participants from different levels of education in terms having positive attitude | | | | |
| --- | --- | --- | --- | --- |
| Level of education | High school or less | Associate degree | Bachelor or professional degree | Postgraduate study or training |
| High school or less |  | 34.8% vs. 65.6% | 34.8% vs. 82.8% | 34.8% vs. 94.3% |
| Associate degree | **<0.0001**^*^ |  | 65.6% vs. 82.8% | 65.6% vs. 94.3% |
| Bachelor or professional degree | **<0.0001** | **0.0005** |  | 82.8% vs. 94.3% |
| Postgraduate study or training | **<0.0001** | **0.0011** | 0.0790 |  |
| Data above the diagonal line represent the comparison in terms of proportions of patients with adequate knowledge in the two categories under investigation. Data beneath the diagonal line represent the *p*-values from the Bonferroni post hoc analysis for each two categories under investigation.  ^*^ *p*-values <0.0083 from Bonferroni post hoc analysis is considered statistically significant, and cells with numbers in bold represent comparisons with significant differences between the two categories under investigation. | | | | |

| Table 6A. Bonferroni post hoc analysis for the adjusted comparison between participants from different levels of education in terms of complying with appropriate practices most of the time | | | | |
| --- | --- | --- | --- | --- |
| Level of education | High school or less | Associate degree | Bachelor or professional degree | Postgraduate study or training |
| High school or less |  | 39.3% vs. 88.9% | 39.3% vs. 88.5% | 39.3% vs. 91.4% |
| Associate degree | **<0.0001**^*^ |  | 88.9% vs. 88.5% | 88.9% vs. 91.4% |
| Bachelor or professional degree | **<0.0001** | 0.9218 |  | 88.5% vs. 91.4% |
| Postgraduate study or training | **<0.0001** | 0.6762 | 0.6048 |  |
| Data above the diagonal line represent the comparison in terms of proportions of patients with adequate knowledge in the two categories under investigation. Data beneath the diagonal line represent the *p*-values from the Bonferroni post hoc analysis for each two categories under investigation.  ^*^ *p*-values <0.0083 from Bonferroni post hoc analysis is considered statistically significant, and cells with numbers in bold represent comparisons with significant differences between the two categories under investigation. | | | | |

| Table 7A. Bonferroni post hoc analysis for the adjusted comparison between medical staff participants from different departments in terms of having adequate knowledge | | | | | |
| --- | --- | --- | --- | --- | --- |
| Department | Medical or surgical | Pharmacy | Laboratory services | Nursing | Other para-clinical services |
| Medical or surgical |  | 85.0% vs. 88.1% | 85.0% vs. 83.6% | 85.0% vs. 64.0% | 85.0% vs. 85.6% |
| Pharmacy | 0.6503^*^ |  | 88.1% vs. 83.6% | 88.1% vs. 64.0% | 88.1% vs. 85.6% |
| Laboratory services | 0.8461 | 0.4661 |  | 83.6% vs. 64.0% | 83.6% vs. 85.6% |
| Nursing | 0.0157 | **0.0011** | 0.0069 |  | 64.0% vs. 85.6% |
| Other para-clinical services | 0.9267 | 0.6414 | 0.7137 | **0.0003** |  |
| Data above the diagonal line represent the comparison in terms of proportions of patients with adequate knowledge in the two categories under investigation. Data beneath the diagonal line represent the *p*-values from the Bonferroni post hoc analysis for each two categories under investigation.  ^*^ *p*-values <0.005 from Bonferroni post hoc analysis is considered statistically significant, and cells with numbers in bold represent comparisons with significant differences between the two categories under investigation. | | | | | |

| Table 8A. Bonferroni post hoc analysis for the adjusted comparison between medical staff participants from different departments in terms of having positive attitude | | | | | |
| --- | --- | --- | --- | --- | --- |
| Department | Medical or surgical | Pharmacy | Laboratory services | Nursing | Other para-clinical services |
| Medical or surgical |  | 90.0% vs. 84.8% | 90.0% vs. 89.6% | 90.0% vs. 58.4% | 90.0% vs. 84.8% |
| Pharmacy | 0.4475^*^ |  | 84.8% vs. 89.6% | 84.8% vs. 58.4% | 84.8% vs. 84.8% |
| Laboratory services | 0.9412 | 0.4188 |  | 89.6% vs. 58.4% | 89.6% vs. 84.8% |
| Nursing | **0.0004** | **0.0007** | **<0.0001** |  | 58.4% vs. 84.8% |
| Other para-clinical services | 0.4068 | 1.0000 | 0.3580 | **<0.0001** |  |
| Data above the diagonal line represent the comparison in terms of proportions of patients with adequate knowledge in the two categories under investigation. Data beneath the diagonal line represent the *p*-values from the Bonferroni post hoc analysis for each two categories under investigation.  ^*^ *p*-values <0.005 from Bonferroni post hoc analysis is considered statistically significant, and cells with numbers in bold represent comparisons with significant differences between the two categories under investigation. | | | | | |

| Table 9A. Bonferroni post hoc analysis for the adjusted comparison between medical staff participants from different departments in terms of complying with appropriate practices most of the time | | | | | |
| --- | --- | --- | --- | --- | --- |
| Department | Medical or surgical | Pharmacy | Laboratory services | Nursing | Other para-clinical services |
| Medical or surgical |  | 82.5% vs. 86.4% | 82.5% vs. 88.1% | 82.5% vs. 97.8% | 82.5% vs. 88.1% |
| Pharmacy | 0.5915^*^ |  | 86.4% vs. 88.1% | 86.4% vs. 97.8% | 86.4% vs. 88.1% |
| Laboratory services | 0.4229 | 0.7853 |  | 88.1% vs. 97.8% | 88.1% vs. 88.1% |
| Nursing | **0.0017** | 0.0073 | 0.0144 |  | 97.8% vs. 88.1% |
| Other para-clinical services | 0.2180 | 0.5019 | 0.7093 | 0.0246 |  |
| Data above the diagonal line represent the comparison in terms of proportions of patients with adequate knowledge in the two categories under investigation. Data beneath the diagonal line represent the *p*-values from the Bonferroni post hoc analysis for each two categories under investigation.  ^*^ *p*-values <0.005 from Bonferroni post hoc analysis is considered statistically significant, and cells with numbers in bold represent comparisons with significant differences between the two categories under investigation. | | | | | |
