# Supplementary material for: Knowledge, Attitude, and Practices Associated With COVID-19 Among Healthcare Workers in Hospitals: A Cross-Sectional Study in Saudi Arabia
Source: Front Public Health. 2021 Jul 23;9:643053. doi: 10.3389/fpubh.2021.643053 (PMC8342857; doi:10.3389/fpubh.2021.643053)
Supplement: Supplementary file 2 [file Data_Sheet_1.PDF]

Appendix A

Invitation to participate in research

The COVID-19 pandemic is the latest threat to humanity, in terms of lives lost and economic break down. As Saudi Arabia battles bravely against this pandemic, medical professionals need to maintain high morale and knowledge about the pandemic if we are to win. In this short survey we aimed to find out the KAP [knowledge, attitude and practices] among health care personnel involved in COVID -19 management in healthcare institutions.

We kindly invite you to fill out this questionnaire, which will be the main pillar in a scientific research entitled "**Knowledge, attitude, and practices associated with COVID-19 among health care workers: A cross-sectional study in Saudi Arabia** ". The study aims to investigate the current level of knowledge, attitude, and practices associated with COVID-19 among health care personnel involved in the management of patients with COVID-19 in Saudi Arabia. Your participation in this questionnaire is optional and all the information collected will be treated in strict confidence. It will take only a few minutes, we sincerely appreciate your time and efforts spent in completing this survey.

Declaration

I have gone through the details of the study and I am well informed about the purpose of this survey. I understand that all of my responses would be maintained strictly confidential, and my participation in this study is purely voluntary. I hereby give my consent to participate in this study.

- ☐ Agree
- ☐ Disagree

The questionnaire contents

First: Elements for the demographic information about the participant

1. Age group:

- ☐ 21 – 30 years
- ☐ 31 – 40 years
- ☐ 41 – 50 years
- ☐ 51 – 60 years
- ☐ 61 – 70 years

2. Gender (Sex):

- ☐ Male
- ☐ Female

3. Current marital status:

- ☐ Married
- ☐ Single

4. Highest educational qualification:

- ☐ High school or less
- ☐ Associate degree
- ☐ Bachelor or professional degree
- ☐ Postgraduate study or training (master, residency, or fellowship)

5. Specialty or service you provide in your hospital:

- ☐ Medical or Surgical
- ☐ Nursing
- ☐ Laboratory services
- ☐ Pharmacists
- ☐ Other paraclinical services
- ☐ Administrative
- ☐ Supporting staff (security, maintenance, and housekeeping)

6. Have you received any infection control training?

- ☐ Yes

- ☐ No

7. What is your main source of information about COVID-19?
   (select all that applies)

- ☐ International health organization websites, like CDC and WHO
- ☐ Ministry of health
- ☐ News media channels
- ☐ Social media

Second: Items related to the main aim of the study

A. Please, answer the following questions regarding your knowledge of COVID-19:

1. COVID-19 is a contagious disease that is caused by:

   - ☐ Fungi
   - ☐ Bacteria
   - ☐ Virus
   - ☐ Parasite
   - ☐ I don't know

2. The most common manifestation for the COVID-19 is:

   - ☐ Cough and fever
   - ☐ Stuffy and runny nose
   - ☐ Mild headache
   - ☐ Abdominal pain and diarrhea
   - ☐ I don't know

3. The disease can easily spread through:

   - ☐ Food
   - ☐ Water
   - ☐ Close contact with animal
   - ☐ Respiratory droplets and close contact
   - ☐ I don't know

4. What is the longest incubation period for COVID-19 before experiencing any symptoms?

   - ☐ 3 days
   - ☐ 5 days
   - ☐ 10 days
   - ☐ 14 days
   - ☐ I don't know

5. Severe cases and death are more common among:

   - ☐ Young children (< 18 years)

- ☐ Youth (19-30 years)
- ☐ Older adults (31-60 years)
- ☐ Elderly (>60 years)
- ☐ I don't know

6. Multiple proven curative treatment options are available now for COVID-19 all over the world:
   - ☐ True
   - ☐ False
   - ☐ I don't know

7. Most COVID-19 cases are mild and can recover with no treatment:
   - ☐ True
   - ☐ False
   - ☐ I don't know

8. We know that the pandemic will be over by summer, as the causative microbe is sensitive to high temperature and humidity:
   - ☐ True
   - ☐ False
   - ☐ I don't know

9. Washing hands with soap and water is effective in eliminating the causative microbe.
   - ☐ True
   - ☐ False
   - ☐ I don't know

B. Attitude: Do you agree or disagree with the following statements:

(Agree – Not Sure – Disagree)

1. In my opinion, all people in the healthcare system and the community are part of this battle against COVID-19, and should be responsible about their role.
2. I believe that early detection of COVID-19 cases through mass testing will facilitate or accelerate the control of the COVID-19 pandemic.
3. I think people who got infected with COVID-19, including health care personnel, were infected due to negligence.
4. I have a feel of threat or fear when I become close or provide care to a confirmed or suspected COVID-19 patient.
5. I think COVID-19 is just a communicable disease which is being given undue importance.
6. I think restricting travels, locking cities, and quarantining all suspected cases are an exaggeration for the current situation.
7. The country’s efforts will succeed in the battle against COVID-19 pandemic.
8. I think when COVID-19 pandemic is over many benefits and good things will be seen.

C. **Practices: Which of the following practises you can say that you are doing most or all of the time:**

(Yes – Not Sure – No)

1. If I or anyone close to me develop any COVID-19 symptoms, I will seek or recommend to others to seek medical attention.
2. When I am putting on the personal protective equipment (PPE), I follow the following order: Suit – Mask – Goggles – Gloves.
3. I have been careful not to carry my mobile phone/pen, etc.… inside the COVID-19 ward.
4. I don’t go out unless it is necessary.
5. When I finish my shift, I dispose the PPE and scrub thoroughly before entering home/quarters.
6. I sanitize my hands with alcohol-based solution before attending to each patient.
7. After using my PPE, I dispose them in the appropriate color-coded bins.
